# Evaluation of Eco-Environmental Quality in the Maceió Metropolitan Region, Alagoas, Brazil

**DOI:** 10.3390/ijerph23050569

**Published:** 2026-04-28

**Authors:** Washington Luiz Félix Correia Filho, José Francisco de Oliveira-Júnior, Dimas de Barros Santiago

**Affiliations:** 1 Instituto de Ciências Atmosféricas (ICAT), Federal University of Alagoas, Av. Lourival Melo Mota, s/n, Maceió 57072-970, AL, Brazil; jose.junior@icat.ufal.br; 2 Programa de Pós-Graduação em Engenharia Civil e Ambiental, Departamento de Engenharia Civil e Ambiental (DECA), Federal University of Paraíba (UFPB), Campus I Cidade Universitária, João Pessoa 58051-900, PB, Brazil; dimas.barros@estudante.ufcg.edu.br

**Keywords:** Greenness, Mann–Kendall, RSEI, Pinheiro, environmental crime

## Abstract

**Highlights:**

**Public health relevance—How does this work relate to a public health issue?**

- Poor environmental quality, resulting from reduction, can intensify the thermal sensation.
- The increase in temperature associated with population densification can trigger cardiovascular problems.

**Public health significance—Why is this work of significance to public health?**

- Population densification worsens environmental quality, which negatively impacts public health.
- The environmental impact in Pinheiro has altered the socioeconomic and health quality of the affected communities.

**Public health implications—What are the key implications or messages for practitioners, policymakers, and/or researchers in public health?**

- The deterioration of environmental quality has led to a decline in the quality of life and health of the population.
- The environmental impact observed in the Pinheiro neighborhood, associated with the replacement of native vegetation by public facilities, has resulted in poorer air quality and increased local thermal sensation for the population.

**Abstract:**

The Maceió Metropolitan Region (MMR) has undergone significant changes due to public policies that promote urban growth. This has intensified environmental impacts, adversely affecting local communities. The Remote Sensing Ecological Index (RSEI), a remote sensing-based metric, was used to evaluate ecosystem quality. The study assessed annual ecosystem quality in the MMR, Alagoas, using RSEI values from MODIS data spanning 2000 to March 2024/2025. To ensure data quality and reliable results, all MODIS data underwent rigorous quality control, including the exclusion of pixels affected by cloud cover, shadows, and missing values. Only data points meeting established MODIS quality assurance standards were used. Annual RSEI values varied considerably, from 0.449 in 2005 to 0.636 in 2014. Most areas in the MMR are classified as moderate quality (0.4 < RSEI < 0.6), particularly in central and eastern sectors. The lowest-quality regions (0 < RSEI < 0.4) are concentrated in the east—including Maceió, the hub city—and the west, largely due to high population density. The Sen-Slope Estimator and trend analysis revealed significant trends in the hub city, with positive trends in the northeast. Urban expansion has led to the loss of native vegetation, including sugarcane fields and remnants of the Atlantic Forest. The Pettitt test identified a structural change in 2018, likely linked to environmental violations related to the Braskem petrochemical industry and salt extraction in Maceió.

## 1. Introduction

In recent decades, Brazilian Metropolitan Regions (BMRs) have undergone profound change; accordingly, authorities have implemented decisive public policies to promote urban development and national expansion [1,2,3,4,5]. Over the past 20 years, targeted programs such as the *Programa de Aceleração do Crescimento* and the *Programa Minha Casa, Minha Vida* have actively spurred urban growth across multiple regions of Brazil [6,7,8].

Government initiatives have accelerated urbanization, directly replacing native vegetation with urban infrastructure and altering the natural environment [6,7,8]. These actions have propelled environmental impacts, resulting in higher air pollution [9] and increased cases of waterborne and arbovirus diseases [10,11]. Reduced vegetation diminishes the ecosystem’s ability to regulate air quality by filtering pollutants and maintaining atmospheric balance, which increases pollution. Furthermore, reduced vegetative cover creates ideal conditions for disease outbreaks by increasing the abundance of vectors—organisms such as mosquitoes that transmit diseases like arboviruses. Remote sensing (RS) products from orbital platforms, such as the Terra satellite, deliver reliable data on Earth’s surface. RS involves acquiring information about Earth’s surface without physical contact, typically via satellite or aerial sensors [12,13].

Remote sensing (RS) directly reveals links among vegetation loss, the expansion of disease vector habitats, and related health outcomes, thereby decisively advancing understanding of the public health effects of urban expansion. RS data robustly assesses ecosystem quality using indices such as the Normalized Difference Vegetation Index (NDVI) [14,15], which measures live green vegetation, as well as urbanization indicators like the Normalized Difference Built-up Index (NDBI) [7,16], which detects built-up areas, and Land Surface Temperature (LST) [8,15,16,17,18], which measures Earth’s surface temperature.

However, prior research has not yet implemented the Remote Sensing Ecological Index (RSEI)—an integrated ecological quality indicator—in Brazil, particularly in BMRs, leaving a clear gap in the literature. The integrated RSEI method employed here addresses this gap and constitutes a notable methodological advance, as these indices are traditionally used alone or alongside other metrics, limiting comprehensive analysis.

The RSEI, originally proposed and developed by Xu [19], integrates several indicators extracted from RS to improve the assessment of ecosystem quality, specifically through the Remote Sensing Ecological Index (RSEI). This index comprises four indicators derived from RS products: Greenness (vegetation), Wetness (humidity), Dryness (urban infrastructure), and Heat (land surface temperature) [12,15,16,19,20,21]. The RSEI uses principal component analysis (PCA) to objectively extract variance related to environmental quality, thereby reducing analyst subjectivity [22].

The RSEI has been used in many regions, especially China [12,14,15,18,19,20,21,23], but its use in Brazil, particularly BMRs, is rare. The Maceió Metropolitan Region (MMR), a rapidly urbanizing coastal city, is a fitting case study. Its demographic and environmental changes mirror trends seen in similar coastal cities globally. The region’s geographic and socio-economic pressures from urban growth and environmental challenges make it relevant for other coastal metropolitan areas. This study evaluates ecosystem quality using MODIS data for the MMR in Alagoas from 2000 to 2025 (as of March).

## 2. Materials and Methods

### 2.1. Study Area

**Figure 1:**
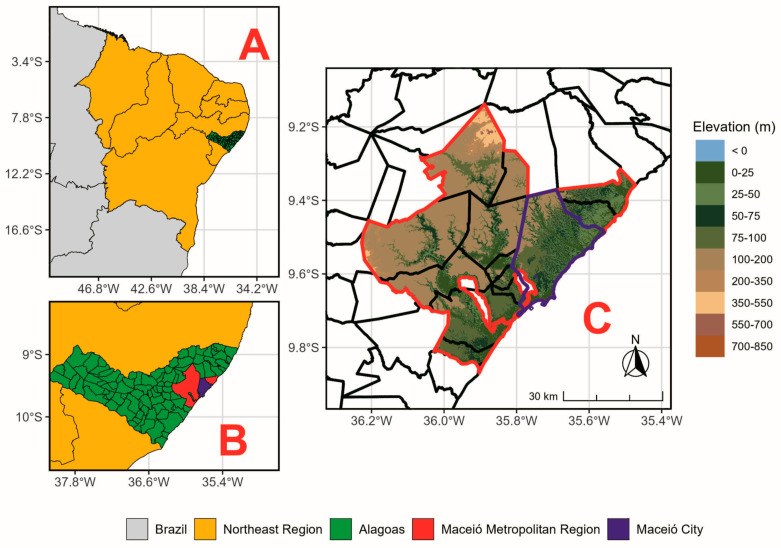
Map of the location of the Maceió Metropolitan Region (MMR), inserted in Brazil (**A**, filled in gray), in the Northeast region (**A**, filled in yellow), in the western part of the state of Alagoas (**B**, filled in green). Elevation map of the MMR (**C**, outlined with a red line) and its hub city, Maceió (outlined with a purple line), with a resolution of 90 m by 90 m, showing elevations ranging from 0 to 850 m. Digital elevation data are provided by the Shuttle Radar Topography Mission (SRTM) database [25].

The RSEI ecosystem quality assessment (ecological and environmental) was decisively conducted in the Maceió Metropolitan Region (MMR), in the eastern part of Alagoas state, Northeast Brazil (Figure 1). This MMR includes 13 municipalities, with an estimated population of 1,328,129 inhabitants and a Gross Domestic Product (GDP) of US$6.258 billion (R$35.423 billion), as outlined in Table 1 [24].

Land-use patterns in these municipalities vary: urban centers are densely populated, while large agricultural areas are dominated by sugarcane [7,8]. Socio-economic contrasts are notable. Some areas undergo rapid urbanization, driving infrastructure development. Others retain rural traits, affecting economics and the environment [7,8].

### 2.2. MODIS Products

For RSEI calculation, we obtained data from the Terra orbital platform using the MODIS sensor, an advanced satellite system that monitors the Earth’s surface. This data was obtained from the National Oceanic and Atmospheric Administration (NOAA) website https://earthexplorer.usgs.gov/ (accessed on 1 March 2025.), which offers valuable resources for environmental analysis and research, as detailed in Table 2.

The RSEI calculation used two MODIS products: (1) MOD09A1.061, which provides measurements of how Earth’s surface reflects sunlight at different wavelengths (surface spectral reflectance bands 1–7) at a 500 m spatial resolution [26]; and (2) MOD11A2.061, which provides temperature information from the thermal band (land surface temperature, LST) at a 1 km resolution [27]. For this evaluation, all data were resampled using nearest-neighbor interpolation, resulting in a spatial resolution of 0.005° × 0.005° (500 m × 500 m).

While this approach allows integration of different data types at a common spatial scale, it is important to note that resampling LST from 1 km to 500 m may introduce spatial inaccuracies, particularly in detecting finer urban features or abrupt transitions between land-cover types. These limitations should be considered when interpreting spatial patterns in highly heterogeneous urban environments.

Nearest-neighbor interpolation preserves the original data values but may introduce blocky artifacts, which can affect heat-island detection. Minimal artifacts were assessed using cross-validation against control datasets to ensure compatibility in scale. Annual spectral band composition occurs from early September to late March each year (spring and summer) in MMR.

### 2.3. Remote Sensing Ecological Index (RSEI)

The Remote Sensing Ecological Index (RSEI) was proposed and developed by [19]. It was later refined by [20] to evaluate ecological and environmental conditions using Earth-orbiting satellite data [15,19,20,28]. This index consists of four environmental indicators: (1) Dryness, (2) Heat, (3) Greenness, and (4) Wetness, which can be expressed as follows:

(1) $$RSEI=f\left(Dryness,Heat,Greenness,Wetness\right)$$

The RSEI was initially developed by [19,20]; however, several studies have adapted or implemented it for local effect diagnosis [18,29,30,31] or have included additional components in their analyses [18,32]. Next, the composition and derivation of each RSEI indicator will be presented, starting with Dryness, which is based on the Normalized Differential Built-up and Soil Index (NDBSI).

#### 2.3.1. Normalized Differential Built-Up and Soil Index (NDBSI—Dryness)

The NDBSI (Normalized Difference Built-up and Soil Index) is a Dryness indicator that detects impervious surfaces resulting from land-cover changes, which can affect ecological patterns [33,34]. It combines two other indices: (1) the Index-based Built-up Index (IBI), which is used for mapping built-up (urban or constructed) areas, and (2) the Soil Index (SI), which is used for identifying bare soil or sparse vegetation, such as that resulting from deforestation. Therefore, these indicators are calculated as follows:

(2) $$IBI\;=\frac{2*\frac{\rho_{SWIR1}}{\left(\rho_{SWIR1}{+\rho }_{NIR}\right)}-\left[\frac{\rho_{NIR1}}{\left(\rho_{NIR1}{+\rho }_{red}\right)}+\frac{\rho_{green}}{\left(\rho_{green}{+\rho }_{SWIR1}\right)}\right]}{2*\frac{\rho_{SWIR1}}{\left(\rho_{SWIR1}{+\rho }_{NIR}\right)}+\left[\frac{\rho_{NIR1}}{\left(\rho_{NIR1}{+\rho }_{red}\right)}+\frac{\rho_{green}}{\left(\rho_{green}{+\rho }_{SWIR1}\right)}\right]}$$

(3) $$SI=\frac{{(\rho }_{SWIR1}{+\rho }_{red})-{(\rho }_{NIR1}{+\rho }_{blue})}{\left(\rho_{SWIR1}{+\rho }_{red}\right)+{(\rho }_{NIR1}{+\rho }_{blue})}$$

where $\rho_{red},\;\;\rho_{green},\;{\;\rho }_{green},\;{\;\rho }_{NIR},\;e\;\rho_{SWIR1}$ correspond to the surface reflectance of the red, green, blue, near-infrared, and mid-infrared bands, respectively. After obtaining the IBI and SI, Dryness is calculated as follows:

(4) $$Dryness\;=\frac{IBI+SI}{2}\;$$

The NDBSI (Dryness) intervals range from −1 (vegetated areas) to 1 (impervious areas).

#### 2.3.2. Normalized Difference Vegetation Index (NDVI—Greenness)

The NDVI is a widely used Greenness indicator for monitoring and detecting vegetation cover at regional and global scales. It evaluates vegetation vigor [15] and detects land-use and land-cover changes in urban areas [7,8,21]. In some studies, the Greenness indicator can be represented by the Enhanced Vegetation Index (EVI) [22,35]. However, in this article, NDVI will be used and is obtained as follows:

(5) $$Greenness=\frac{\rho_{NIR1}-\rho_{red}}{\rho_{NIR1}+\rho_{red}}$$

where $\rho_{red}\;e\;\rho_{NIR}$ correspond to the surface reflectance of the red and near-infrared bands, respectively; the NDVI (Greenness) range extends from −1 (indicating water presence) to 1 (indicating dense vegetation).

#### 2.3.3. Land Surface Temperature (LST—Heat)

LST represents the Heat indicator, which assesses the effects of urbanization on ecological processes through land-use and cover change and soil temperature [8,9,17,18]. To obtain the LST, data from the 6th version of MOD11A2 on the Terra satellite are used [26], starting from the digital number (ND):

(6) $${LST}_{0}\;(in\;Kelvin)\;=0.02\times ND$$

(7) $$Heat\;(LST\;converted\;in\;Celsius,\;{}^{\circ}C)={LST}_{0}-273.15$$

LST refers to land surface temperature in Kelvin, while ND is the pixel’s digital number. The Heat component is initially provided in Kelvin and later converted to °C.

#### 2.3.4. Tasseled Cap (Wetness)

The Tasseled Cap transformation indicates the Wetness component, which reflects the physical parameters of the surface–atmosphere interface, specifically soil moisture conditions [34,35,36]. This method is widely employed in environmental studies [22,28,33,37]. The formulas for deriving it via MODIS are as follows:

(8) $$\begin{array}{cc} Wetness= & 0.1147\rho_{red}+0.2489\rho_{NIR1}+0.2408\rho_{blue}+0.3132\rho_{green}\\ & \;\;\;\;\;\;+0.3122\rho_{NIR2}-0.6416\rho_{SWIR1}-0.5087\rho_{SWIR2} \end{array}$$

where $\rho_{red},\;{\;\rho }_{blue},\;\;\rho_{green},\;\;\rho_{NIR},\;\;e\;\rho_{SWIR1},\;\;e\;\rho_{SWIR2}$ correspond to the surface reflectance of the red, green, blue, near-infrared, and mid-infrared 1 and 2 reflectance bands, respectively.

#### 2.3.5. Obtaining the RSEI

To calculate the RSEI, each of the four indicators (Dryness, Greeness, Heat, and Wetness) was first normalized separately to remove differences or outliers related to the indicators’ units, as illustrated by Equation (9):

(9) $${NI}_{i}=\frac{I_{i}-I_{min}}{I_{max}+I_{min}}$$

where ${NI}_{i}$ is the normalized value indicator i (which can be Dryness, Greeness, Heat, and Wetness), $I_{i}$ is the initial value of indicator i, and $I_{max}$ and $I_{min}$ are the maximum and minimum values of indicator i, respectively.

The next step after normalizing the indicators is to perform principal component analysis (PCA) on the normalized indicators to extract the first component that accounts for the largest proportion of the explained variance. If the Greenness and Wetness indicators are less than 0, we subtract 1 from the obtained PC1, as described below:

(10) $${RSEI}_{0}=\left\{\begin{array}{c} if\;the\;{Greenness}_{n}\;and\;{Wetness}_{n}>0,\\ PC1\;\left({Dryness}_{n},\;{Heat}_{n},{Greenness}_{n},\;{Wetness}_{n}\right),\;\\ {if\;the\;Greenness}_{n}\;and\;{Wetness}_{n}<0,\\ \;1-PC1\;({Dryness}_{n},\;{Heat}_{n},\;{Greenness}_{n},\;{Wetness}_{n})\; \end{array}\right.$$

After obtaining the ${RSEI}_{0}$, the normalization calculation in the RSEI was performed:

(11) $${RSEI}_{n}=\frac{{RSEI}_{0}-{RSEI}_{min}}{{RSEI}_{max}+{RSEI}_{min}}\;$$

${RSEI}_{n}$ should vary between 0 and 1 [15,33]. Where ${RSEI}_{0}$ is the ecological and environmental index of the evaluated year (for the consideration of a year involves the following year; for example, the year 2001 covers the period from September 2000 to March 2001, and this pattern continues up to 2025, which spans from September 2024 to March 2025), ${RSEI}_{max}\;and\;{RSEI}_{min}$ are the minimum and maximum RSEI values for each analyzed pixel, and ${RSEI}_{n}$ is a normalized RSEI.

${RSEI}_{n}$ values range from 0 to 1 and can be classified as follows: (a) Poor, between 0 and 0.2; (b) Fair, between 0.2 and 0.4; (c) Moderate, between 0.4 and 0.6; (d) Good, between 0.6 and 0.8; and (e) Excellent, between 0.8 and 1. These class thresholds are based on standards established in previous research for ecological quality assessment using RSEI, which divide the index range into equal intervals to simplify interpretation and improve comparability across studies [15,33]. Such intervals help distinguish regions with severe ecological degradation from those with better environmental conditions and are widely used in studies applying the RSEI methodology to urban and regional landscapes.

After obtaining the annual thematic maps from MODIS data, the median Theil-Sen Slope statistical trend and Pettitt tests will be used to assess whether the temporal evolution of the RSEI in the MMR is statistically significant.

### 2.4. Theil-Sen Estimator and Mann–Kendall Test

The evaluation of the average trend using the Theil-Sen estimator, also known as the Sen-Slope Estimator, is a robust nonparametric method that calculates the overall trend from data collected over extended periods [38,39,40]. This approach will determine whether the RSEI quality indicates a statistically significant worsening or improvement trend in MMR areas. Additionally, this method is resistant to measurement errors or outliers [37,39,40]. To evaluate the magnitude of the trend, we will use Sen’s slope estimator (β) obtained from the following expression:

(12) $$\beta =mean\left(\frac{{RSEI}_{j}-{RSEI}_{i}}{j-i}\right)$$

where $i>j\;(2001\leq i\leq j\leq 2025),\;\;{RSEI}_{j}\;and\;{RSEI}_{i}$ are RSEI values of years i and j, respectively. Keep in mind that these years refer to the period from September of the year before the current March through March of the current year; for example, the year 2001 includes September 2000 through March 2001.

When β>0 (β<0), it refers to the trend of the RSEI pixel increasing or decreasing. After calculating the median Theil-Sen trend slope, the Mann–Kendall statistical test (S) is performed [41,42]:

(13) $$S=\sum_{i=1}^{n=1}\sum_{j=i+1}^{n}sign\left({RSEI}_{j}-{RSEI}_{i}\right)\;$$

(14) $$Sign({RSEI}_{j}-{RSEI}_{i})=\left\{\begin{array}{c} +1\;\left(if\;{RSEI}_{j}-{RSEI}_{i}>0\right)\\ \;0\;\left({if\;RSEI}_{j}-{RSEI}_{i}=0\right)\\ -1\;\left(if\;{RSEI}_{j}-{RSEI}_{i}<0\right) \end{array}\right.$$

After performing the Mann–Kendall statistical test, the trend was further analyzed using the Z statistical test, which was obtained as follows:

(15) $$Z=\;\left\{\begin{array}{c} \frac{S-1}{\sqrt{Var\left(S\right)}},\;if\;S>0\\ 0\;,\;if\;S=0\\ \frac{S+1}{\sqrt{Var\left(S\right)}},\;if\;S<0 \end{array}\right.$$

(16) $$Var\left(S\right)=\frac{1}{n}\left[n\left(n-1\right)(2n+5)\right]\;$$

where Z represents the value from the statistical significance test calculated for each pixel RSEI $\left(-\infty ,+\infty \right)$, and n is the number of years in the 25-year time series (September 2000–March 2001 as year 2001, until September 2024–March 2025 as year 2025).

In this study, S is the statistical measure used to assess the presence of a trend in the annual RSEI time series. The significance level used will be $\alpha =0.10\;(\left|Z\right|>\;1.645),\;0.05\;(\left|Z\right|>\;1.96$), and 0.01 $(\left|Z\right|>\;2.574$), that is, a 90%, 95% and 99% confidence level, respectively. The two-tailed test compares the calculated Z value with critical values. Exceeding this limit indicates that the RSEI value is statistically significant [37,39,40].

### 2.5. Change Points Structure Detection (Pettitt Test)

The Pettitt method is a rank-based, nonparametric statistical test that detects breakpoints in a series, indicating the exact point at which a trend shifts if one occurs [43]. As a result, this test involves partitioning the original non-stationary series into two stationary series with different means and distributions [9,44]. The null hypothesis (H0) assumes there is no breakpoint in the series, while the alternative hypothesis (H1) suggests that a breakpoint exists. Pettitt’s method is a variation in the Mann–Whitney test, where the identification of a change point, a statistical index, is defined as follows [9,43]:

(17) $$U_{t,T}=\sum_{i=1}^{t}\sum_{j=i+1}^{T}sgn\left(x_{j}-x_{i}\right),\;1\leq t<T$$

where

(18) $$Sign\;(\theta )=\left\{\begin{array}{c} +1\;\left(if\;x>0\right)\\ \;0\;\left(if\;x=0\right)\\ -1\;\left(if\;x<0\right) \end{array}\right.$$

when the series follows a continuous distribution, the test statistics $U_{t,T}$ can also be obtained using the following recursive relation:

(19) $$U_{t,T}=U_{t-1,T}+V_{t,T}\;$$

For t=1,…, T, where

(20) $$V_{t,T}=\sum_{j=1}^{T}sgn\left(x_{j}-x_{i}\right)\;$$

The most probable change point τ is found where its value satisfies

(21) $$K_{\tau }=\left|U_{\tau ,T}\right|=max\left|U_{t,T}\right|$$

The significance probability associated with the value is approximately evaluated as in Equation (22):

(22) $$P=\;2\;{exp}^{\frac{-6k^{2}}{\left(r^{2}+r^{2}\right)}}$$

Given a significance level α, if p<α, we reject the null hypothesis and conclude that $x_{t}$ is a significant change point at level α. In this case, we used the α = 0.10. Conversely, if the *p*-value exceeds 0.10, there is not enough evidence to suggest a trend in the RSEI time series [45].

All procedures, including data extraction, resampling, calculation of monthly averages, and conversion to seasonal and annual averages, the results of the Mann–Kendall and Pettitt tests, and the creation of thematic maps, are carried out using R version 4.4-3 [46].

## 3. Results

The following results will address the annual variability of RSEI and its changes over time, based on the MK and Pettitt tests, as well as the components that make it up (Dryness, Greenness, Heat, and Wetness), to provide a better spatial understanding of the environmental quality of MMR.

### 3.1. Annual RSEI Components

**Figure 2:**
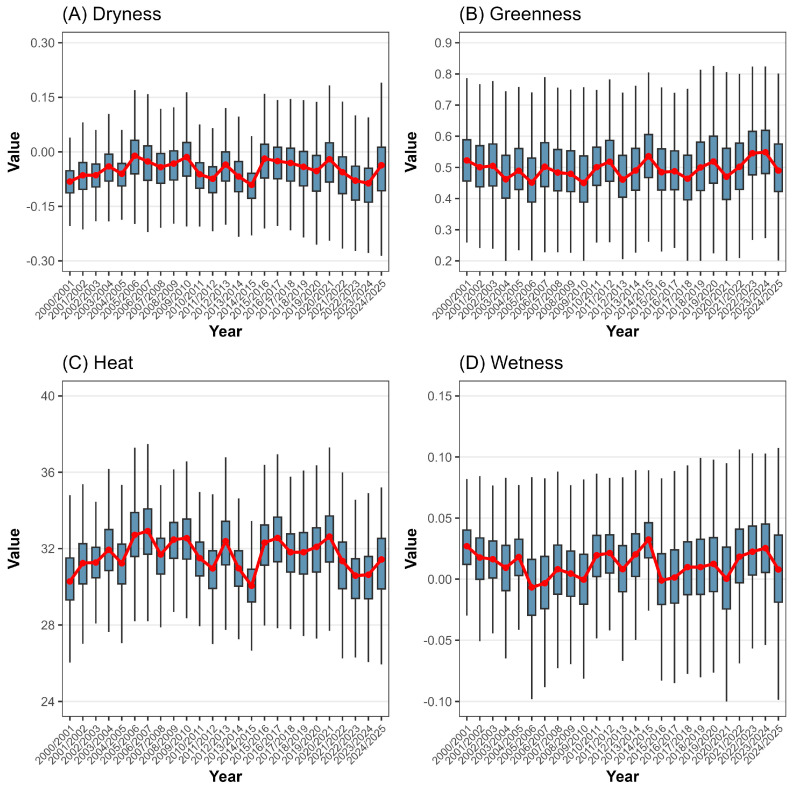
Annual RSEI components boxplot ((**A**)—Dryness, (**B**)—Greenness, (**C**)—Heat, (**D**)—Wetness) from 2000/2001 to 2024/2025 for the MMR, Alagoas, Brazil. The red line indicates the annual mean. Keep in mind that these years refer to the period from September of the year before the current March to March of the current year; for example, the year 2001 includes September 2000 through March 2001. Data sources: [26,27].

Figure 2 illustrates the annual variations in the four RSEI components: Dryness (Figure 2A), Greenness (Figure 2B), Heat (Figure 2C), and Wetness (Figure 2D). The annual patterns show similarities between components with anthropic characteristics (Dryness and Heat), linked to urban growth, and those with environmental characteristics (Greenness and Wetness). Notably, Dryness and Heat show peaks followed by declines over the next two years, with both decreasing noticeably from 2015/2016 to 2023/2024. Conversely, variability in Greenness and Wetness increases progressively within the same period. On average, Dryness ranges from −0.09 in 2023/2024 to −0.02 in 2005/2006, while Heat ranges from 30.41 °C in 2000/2001 to 32.81 °C in 2006/2007. Greenness consistently exceeds 0.40, from 0.462 (2005/2006) to 0.546 (2023/2024). Wetness fluctuates between −0.01 in 2005/2006 and 0.027 in 2014/2015.

**Figure 3:**
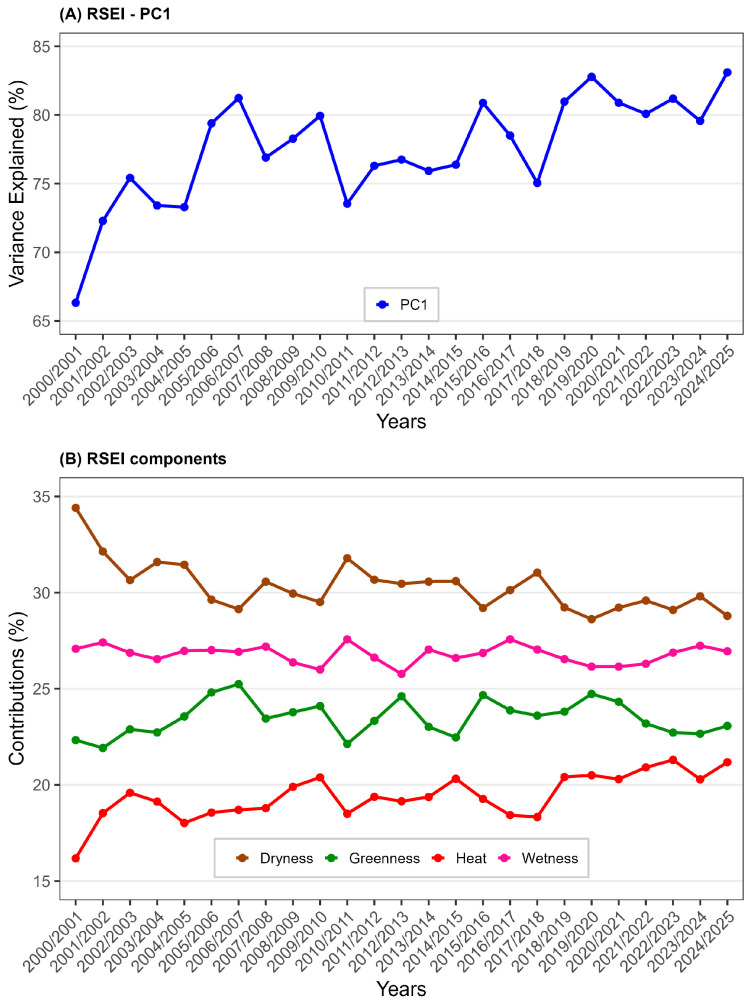
Time series of the First Principal Component (PC1) of the RSEI (shown as a blue line in (**A**)) and the percentage contribution of each component (represented by colors in (**B**)) that compose the RSEI—Dryness (brown), Greenness (green), Heat (red), and Wetness (pink)—from 2000/2001 to 2024/2025 for the MMR, Alagoas, Brazil. Keep in mind that these years refer to the period from September of the year before the current March to March of the current year; for example, the year 2001 includes September 2000 through March 2001. Data sources: [26,27].

Figure 3 illustrates the annual fluctuations of the first principal component (PC) of the RSEI (Figure 3A) and its components (Figure 3B) from 2000/2001 to 2004/2025. The analysis of the RSEI PC1 explained variance (Figure 3A) reveals a steady increase, indicating that the components influencing the index’s composition increasingly account for variability over time. Notably, the explained variance increases from 66.33% to approximately 83.10%, thereby enhancing the understanding of variability in ecosystem quality. Apart from the early years (2000/2001 to 2004/2005), the years 2010/2011 and 2017/2018 show explained variance values near or below 75%. When examining the contribution of the four components of the RSEI, Dryness contributes most significantly (with a minimum of 28.79% to a maximum of 34.41%), followed by Wetness (with a minimum of 25.77% to a maximum of 27.57%), Greenness (with a minimum of 21.92% to a maximum of 25.24%), and Heat, which accounts for less than 22% (with a minimum of 16.18% to a maximum of 21.30%).

### 3.2. Annual RSEI Pattern

**Figure 4:**
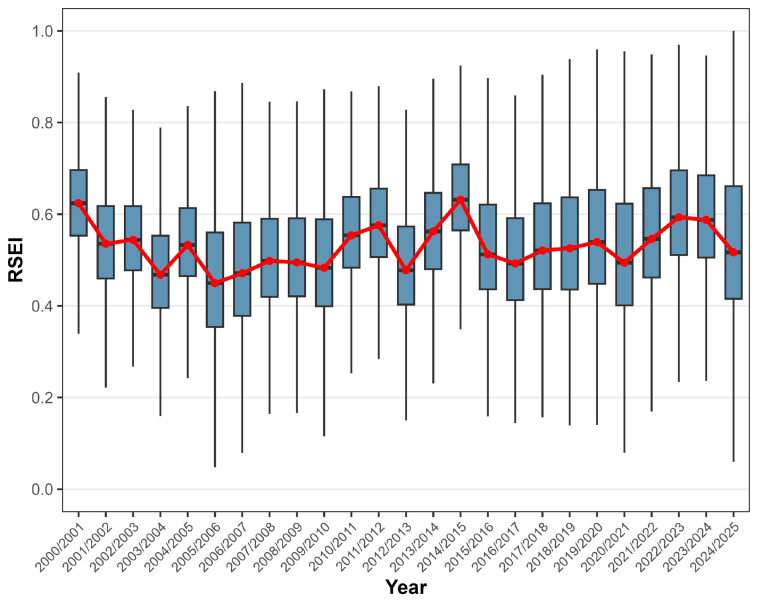
Annual RSEI boxplot from 2000/2001 to 2024/2025 for the MMR, Alagoas, Brazil. The red line indicates the annual mean. Keep in mind that these years refer to the period from September of the year before the current March to March of the current year; for example, the year 2001 includes September 2000 through March 2001. Data sources: [26,27].

Firstly, it was necessary to analyze the annual RSEI pattern using a boxplot (Figure 4) and present its descriptive statistics in Table 3. It is evident that the RSEI fluctuates annually between 0.449 (2005) and 0.636 (2014), with annual amplitudes of 0.06 and 0.96 in 2024 and 2019, respectively. The coefficient of variation (CV) varies from 16.733% (2014) to 32.028% (2005), indicating substantial heterogeneity in RSEI values across the MMR. Comparing these CV values with established ecological resilience thresholds for Atlantic Forest fragments may provide further context, facilitating the interpretation of statistical metrics in ecological terms.

**Figure 5:**
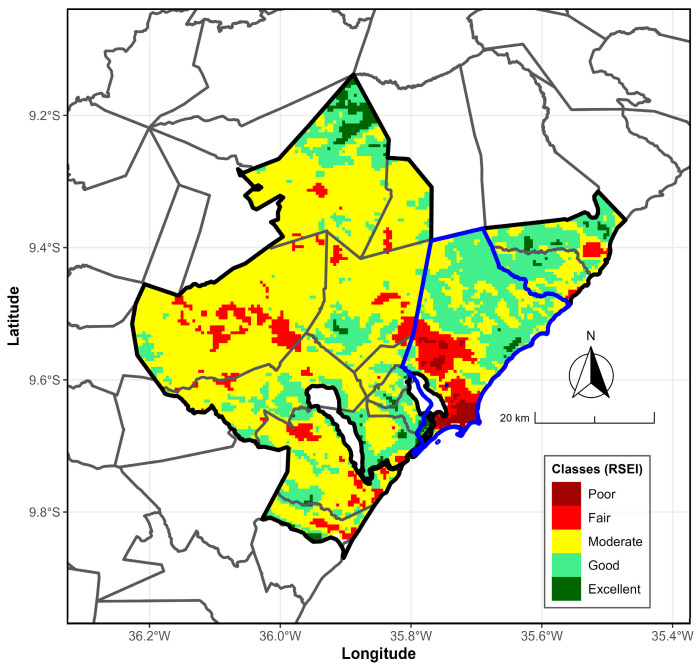
Average annual RSEI map from 2000/2001 to 2024/2025 for the MMR, Alagoas, Brazil. The RSEI map (colors) is classified as follows: Poor (darkred), Fair (red), Moderate (yellow), Good (lightgreen), Excellent (darkgreen). The black and blue boundary lines mark the MMR and hub city, Maceió, respectively.

According to Figure 5, which shows the average annual RSEI map for the period 2000/2001–2024/2025, most of the MMR falls within the moderate class (0.4 < RSEI < 0.6), supporting the observations from the annual boxplots (Figure 4). These RSEI values are primarily found in the region’s central and eastern parts. Additionally, areas with good environmental quality (0.6 < RSEI < 0.8) are located in the central and southern parts, as well as toward the northeast. The excellent class appears in the far north of the MMR. In contrast, regions with poorer environmental conditions, classified as reasonable (0.2 < RSEI < 0.4), are scattered across the eastern and western areas. The poor class (RSEI < 0.2) is only present in the west; both classes are concentrated in the south and southwest of the metropolitan hub city, Maceió.

### 3.3. Annual RSEI Trend Analysis

**Figure 6:**
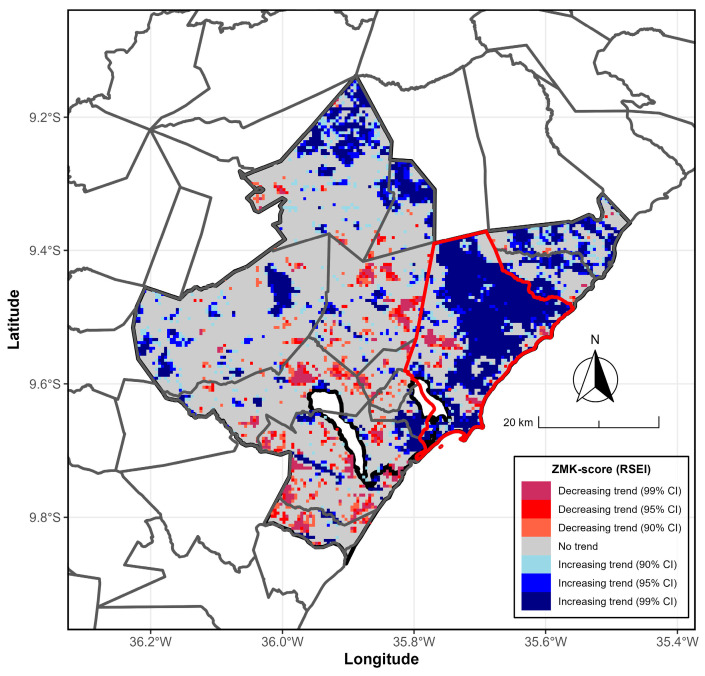
Annual RSEI Mann–Kendall trends from 2000/2001 to 2024/2025 for the MMR in Alagoas, Brazil. ZMK scores filled with maroon, red, and tomato (with lightblue, blue, and navy) denote decreasing (or increasing) trends at 90%, 95%, and 99% confidence intervals (CIs), respectively. Areas filled with gray indicate no observable trend. The gray and red boundary lines mark the MMR and the hub city, Maceió, respectively. Data sources: [26,27].

**Figure 7:**
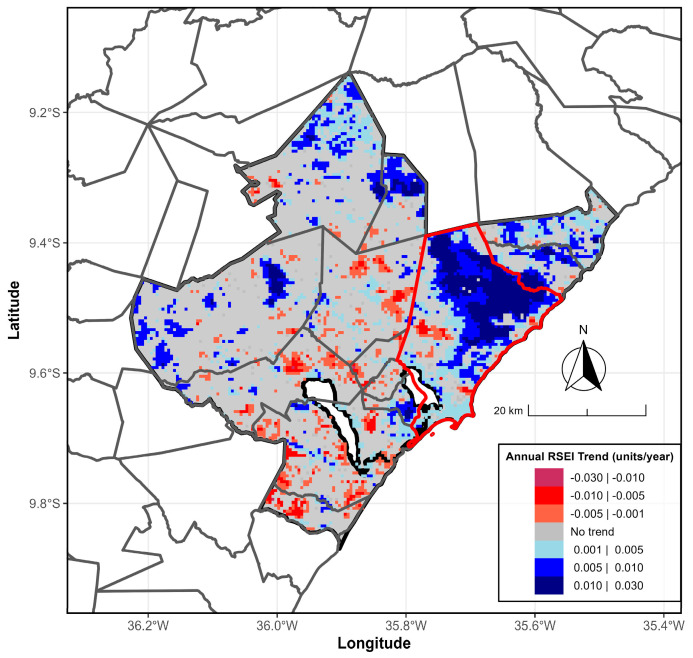
Annual RSEI Sen-Slope Estimator trend from 2000/2001 to 2024/2025 for the MMR, Alagoas, Brazil. Trends range from −0.03 year^−1^ (maroon, red, and tomato colors) to +0.03 year^−1^ (lightblue, blue, and navy colors). Areas filled with gray indicate no observable trend. The gray and red boundary lines mark the MMR and the hub city, Maceió, respectively. Data sources: [26,27].

The following results correspond to the Mann–Kendall Z-score (Figure 6) and the Sen-Slope Estimator (Figure 7) for the annual RSEI from 2000/2001 to 2024/2025. A significant portion of the MMR is not statistically significant (*p* > 0.10). Conversely, a large part of the hub city (Maceió) shows statistical significance (*p* < 0.10). The Z-score map of the Mann–Kendall test for the annual RSEI indicates that, in much of the Maceió City region, there is an increasing trend at the 90% and 95% confidence interval (CI), respectively, mainly observed in Maceió City and the northern sector. Meanwhile, the decreasing trend at the 95% and 99% CI, respectively, occurs sporadically in the central and southern regions of the MMR.

When analyzing the annual RSEI Sen-Slope Estimator trend along the MMR (Figure 7), positive trends are predominant, with values ranging from 0.005 to 0.030. Conversely, the southern part of the city mainly shows positive trends. These results demonstrate the average annual variability of the RSEI and its temporal changes, as identified by the MK and Pettitt tests, and offer a spatial perspective on environmental quality in the MMR.

### 3.4. Annual Change Points Detection

**Figure 8:**
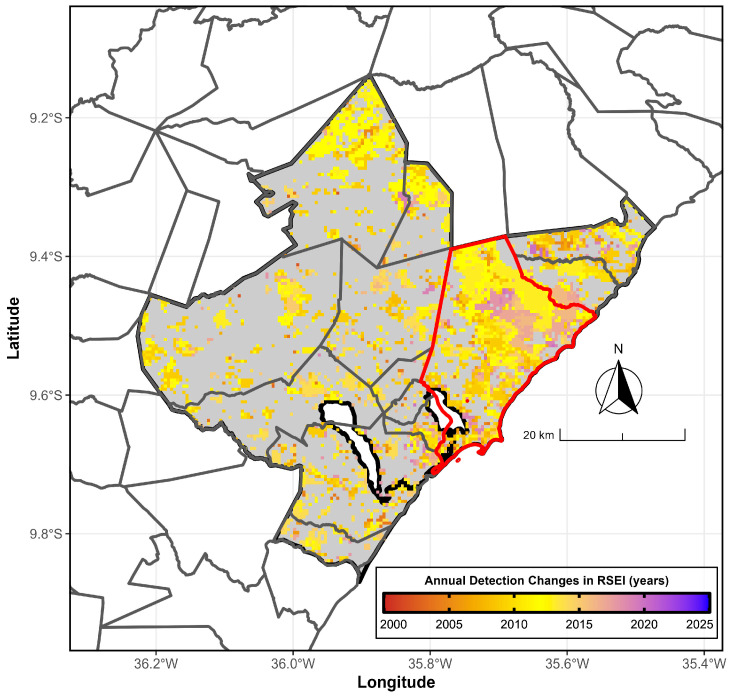
Structural change points (years) of the RSEI based on the Pettitt test from 2000/2001 to 2024/2025 for the MMR, Alagoas, Brazil. Structural changes identified by the Pettitt test (color) occur in 2001 (succubus, beginning), 2012 (yellow, middle of the series), and 2025 (blue, end of the series). The filled gray area is not statistically significant (*p* > 0.10). The gray and red boundary lines mark the MMR and the hub city, Maceió, respectively. Data sources: [26,27].

Figure 8 shows the detection of the yearly change point in the RSEI using the Pettitt test for the period 2000/2001–2024/2025. Regions with significant trends experienced these changes at varying times. Notably, these trend regions appeared between 2008/2009 and 2012/2013, and again between 2015/2016 and 2018/2019, mainly in the northeastern part of the hub.

## 4. Discussion

Based on the RSEI environmental quality, a degraded area is visible in the southern part of Maceió metropolitan area (Figure 5, marked in red), specifically in the city of Maceió, the capital of Alagoas. The annual average pattern distinctly highlights the city center, with RSEI scores categorized as poor (below 0.2) and reasonable (0.2–0.4). This environmental decline is mainly due to the high population density—around 953,000 residents—which significantly pressures the real estate market to fulfill the housing shortage.

Another concern related to the impact of this land-use change and occupation—driven by unplanned urban expansion—is the gradual loss of native vegetation and the replacement of sugarcane cultivation with human-made infrastructure, such as housing complexes, commercial areas, and roads. This shift contributes to higher LST, as shown in multiple studies such as [2,7,8,35,37].

The annual average pattern clearly delineates the city center, with RSEI values classified as poor (RSEI < 0.2) and reasonable (RSEI: 0.2–0.4). This low environmental quality is primarily attributed to high population density (approximately 953,000 inhabitants), which places significant pressure on the real estate sector to address the housing deficit.

These studies have examined the relationship between urban expansion in Maceió and changes in LST over various periods. The findings reveal a substantial increase in LST over the past 40 years, with values exceeding 30 °C in certain areas of the hub city [8]. Additionally, significant changes in land use and occupation have been observed, particularly in the northeastern and northern regions. These observations are consistent with the RSEI trend identified through the Mann–Kendall test, indicating that urban growth is negatively impacting environmental quality in the central and northeastern areas of Maceió.

The Pettitt test also identified a structural change in the time series in 2018 in the hub city (northeastern portion), Maceió, associated with an environmental crime committed by the multinational company Petrochemical Braskem Industries, resulting from rock salt extraction [47]. This incident displaced approximately 60,000 residents from five neighborhoods (Bebedouro, Bom Parto, Farol, Mutange, and Pinheiro), significantly disrupting urban expansion in the city.

## 5. Conclusions

The analysis of environmental quality evolution using the RSEI in the Maceió metropolitan region revealed several key findings. After 2018, a gradual decline in environmental quality was observed, particularly in the capital city, Maceió. This decline is partially attributed to increased real estate speculation in recent years, especially in the northwest and northern sectors of Maceió, driven by high population density and ineffective public policies related to the MMR Master Plan. Additionally, an environmental crime in 2018, resulting from rock salt extraction by the multinational petrochemical company Braskem, affected the neighborhoods of Bebedouro, Bom Parto, Mutange, Pinheiro, and Farol.

These results highlight the urgent need for more effective urban and environmental policy interventions to protect remaining native vegetation, improve land-use planning, and enforce stronger environmental regulations. Policymakers should consider integrating remote sensing monitoring, such as RSEI, into urban management frameworks to promptly detect adverse ecological trends and support decision-making. Future research could expand on these findings by incorporating socio-economic indicators or higher-resolution satellite data to further refine the detection of environmental change and guide targeted mitigation strategies in rapidly growing metropolitan regions.

## Figures and Tables

**Table 1:** List of municipalities that compose the Maceió Metropolitan Region, Alagoas, Brazil, with a total population based on the 2022 Census [24].

Municipality	Population	Municipality	Population
Atalaia	37,512	Murici	25,187
Barra de Santo Antônio	16,365	Paripueira	60,370
Barra de São Miguel	7944	Pilar	16,365
Coqueiro Seco	5581	Rio Largo	93,927
Maceió	957,916	Santa Luzia do Norte	6919
Marechal Deodoro	60,370	Satuba	24,278
Messias	15,405	**Total**	**1,328,129**

**Table 2:** Details on MODIS products and their spatial and temporal resolutions.

Products (Unit)	Spatial and Temporal Resolution	References
MOD09A1.061	500 m × 500 m/8 days	[26]
MOD11A2.061	1000 m × 1000 m/8 days	[27]

**Table 3:** Annual RSEI descriptive statistics. Min—minimum, Q1—1st quartile, Mean—median, Q3—3rd quartile, Max—maximum, SD—standard deviation, CV—coefficient of variation.

Year	Min	Q1	Mean	Median	Q3	Max	SD	CV
2001	0.222	0.462	0.542	0.538	0.619	0.856	0.114	20.947
2002	0.267	0.481	0.550	0.545	0.616	0.828	0.104	18.891
2003	0.160	0.396	0.476	0.468	0.551	0.789	0.115	24.197
2004	0.242	0.468	0.542	0.534	0.613	0.836	0.108	19.968
2005	0.048	0.354	0.460	0.449	0.559	0.868	0.147	32.028
2006	0.079	0.378	0.483	0.471	0.580	0.886	0.144	29.737
2007	0.164	0.421	0.507	0.498	0.589	0.845	0.123	24.374
2008	0.166	0.423	0.507	0.495	0.588	0.846	0.127	25.016
2009	0.115	0.399	0.496	0.482	0.586	0.873	0.137	27.730
2010	0.253	0.487	0.563	0.555	0.636	0.868	0.113	20.006
2011	0.284	0.510	0.583	0.577	0.655	0.879	0.109	18.653
2012	0.147	0.404	0.490	0.478	0.572	0.828	0.123	25.149
2013	0.231	0.485	0.566	0.565	0.647	0.895	0.120	21.246
2014	0.349	0.573	0.641	0.636	0.711	0.924	0.107	16.733
2015	0.159	0.437	0.528	0.512	0.617	0.897	0.137	25.938
2016	0.144	0.414	0.502	0.492	0.588	0.859	0.131	26.162
2017	0.157	0.438	0.531	0.522	0.623	0.904	0.138	25.886
2018	0.139	0.437	0.538	0.526	0.636	0.939	0.148	27.538
2019	0.140	0.450	0.553	0.540	0.654	0.960	0.148	26.775
2020	0.079	0.401	0.513	0.494	0.622	0.956	0.161	31.318
2021	0.170	0.463	0.562	0.547	0.657	0.948	0.141	25.090
2022	0.234	0.516	0.607	0.596	0.698	0.970	0.133	21.975
2023	0.236	0.509	0.599	0.590	0.686	0.946	0.127	21.252
2024	0.060	0.415	0.542	0.517	0.661	1.000	0.167	30.831

## Data Availability

The datasets generated and/or analyzed during the current study are available from the corresponding author upon reasonable request.
